# PET/CT imaging in management of concomitant Hodgkin lymphoma and tuberculosis – a problem solver tool

**DOI:** 10.1002/ccr3.1248

**Published:** 2017-11-29

**Authors:** Nina Jehanno, Thibaut Cassou‐Mounat, Anne Vincent‐Salomon, Marie Luporsi, Manel Bedoui, Frédérique Kuhnowski

**Affiliations:** ^1^ Department of Nuclear Medicine Institut Curie Paris France; ^2^ Department of Pathology Institut Curie Paris France; ^3^ Department of Hematology Institut Curie Paris France

**Keywords:** ^18^F‐FDG, Hodgkin lymphoma, PET/CT, tuberculosis

## Abstract

Infectious lymph nodes mimicking lymphoma is challenging for accurate staging. Although ^18^F‐FDG is a nonspecific tracer accumulating not only in tumor cells but also in inflammatory tissues, the metabolic features and uptake kinetics give valuable information: ^18^F‐FDG PET/CT appears as a useful problem solver tool in ambiguous situation.

## Introduction

Initial accurate staging is crucial for optimal lymphoma treatment planning 1. Overlap of different diseases can be complex for management, in particular overlap of conditions involving lymph nodes extension.

## Case Report

We report the case of a 28‐year‐old man, with no previous history, addressed for the management of suspect Hodgkin lymphoma. He presented with an isolated progressively growing cervical tumefaction, ECOG1, and no night sweats. Cervical lymph node biopsy revealed Hodgkin scleronodular lymphoma with Hodgkin Reed Sternberg cells, CD30 and CD15 positive. Thorax X‐ray and CT scan revealed an alveolo‐interstitial syndrome, suspected to be tuberculosis. Bronchoscopy was noncontributive but bronchoalveolar lavage became positive for mycobacterium tuberculosis.

**Figure 1:**
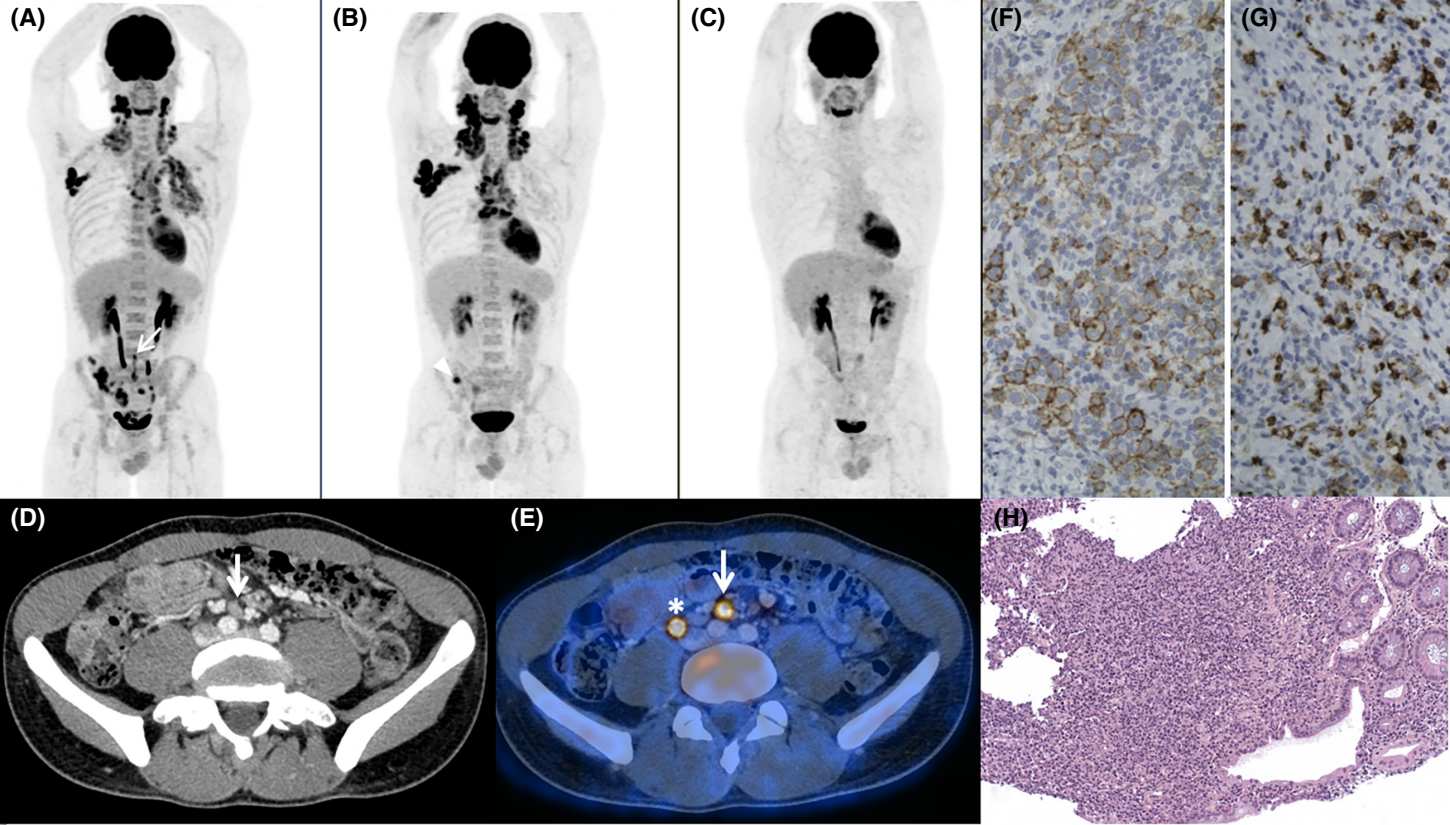
(A) ^18^F‐FDG PET/CT MIP (maximum intensity projection) image of initial Hodgkin lymphoma staging showing intense hypermetabolic uptake of cervical, right axilla and mediastinum lymph nodes, pulmonary and colon uptake, with below diaphragm lymph nodes (white arrow). (B) PET/CT MIP image after 4 weeks of anti‐TB treatment, with complete metabolic response of below diaphragm lymph nodes and persistence of focal colon uptake (arrow head) and partial response of pulmonary TB disease. Note that there is a slightly increase in HL above diaphragm lymph nodes (treatment pending). (C) End‐of‐HL‐treatment (4 ABVD) PET/CT with complete metabolic response. (D) CT scan and fused PET/CT (E) of initial staging with hypermetabolic right iliac primitive lymph node (with arrow), note physiological urinary excretion (*). (F) Immunostaining of cervical lymph node biopsy with an anti‐CD30 antibody (Clone BerH2‐Dako). Hodgkin cells showed a membranous and intracytoplasmic positive staining. (G) Immunostaining of cervical lymph node biopsy with an anti‐CD15 antibody (Clone Carb3‐Dako). Intracytoplasmic positivity of the Hodgkin cells together with the neutrophils. (H) Colonic biopsy revealing tuberculosis with granuloma with epithelioid cells and giant cells.

^18^F‐FDG PET/CT scan was performed for initial staging of his lymphoma confirming intense hypermetabolic cervical lymph node involvement. There was an increased uptake of the alveolo‐interstitial syndrome associated with loco‐regional mediastinal lymph nodes. In addition, PET depicted an incidental digestive colon intense uptake, with below diaphragm lymph nodes. Colonic biopsy was therefore performed, revealing digestive colon tuberculosis localization. Staging was thus equivocal on account of the overlap of both tuberculosis (TB) and Hodgkin lymphoma (HL): stage II HL with only above diaphragm lymph nodes, associated with pulmonary and colic tuberculosis with loco‐regional TB lymph nodes, or stage III HL with above and below diaphragm lymph nodes, associated with pulmonary and colic tuberculosis. Case was discussed among multidisciplinary staff. Due to hemorrhage risk, biopsy of pericolonic lymph nodes was not achievable and empiric quadruple drug regimen therapy for tuberculosis was first initiated (isoniazid, rifampin, pyrazinamide, and ethambutol) while HL treatment is pending. As to solve the Hodgkin lymphoma staging issue, an early PET/CT evaluation was performed after 1‐month antituberculosis therapy. PET/CT showed partial metabolic response of pulmonary and colic tuberculosis localization and complete metabolic response of pericolonic below diaphragm lymph nodes, in favor of their tuberculosis origin. Patient was therefore staged IIAa for Hodgkin disease, unfavorable group (>4 lymph nodes groups), and specific treatment was initiated consisting of chemotherapy with ABVD (doxorubicin–bleomycin–vinblastine–dacarbazine) and radiation therapy (30 Gy). Final PET/CT assessment after 4 ABVD showed complete metabolic response of lymphoma (Deauville 2 criteria) 2, 3 and complete disappearance of tuberculosis (Fig. 1). Accurate staging allowed avoiding overtreatment with BEACOPP and the known infectious risks especially with the indication of TB, and this early stage of disease benefited from complementary radiotherapy planning 4.

## Discussion

**Figure 2:**
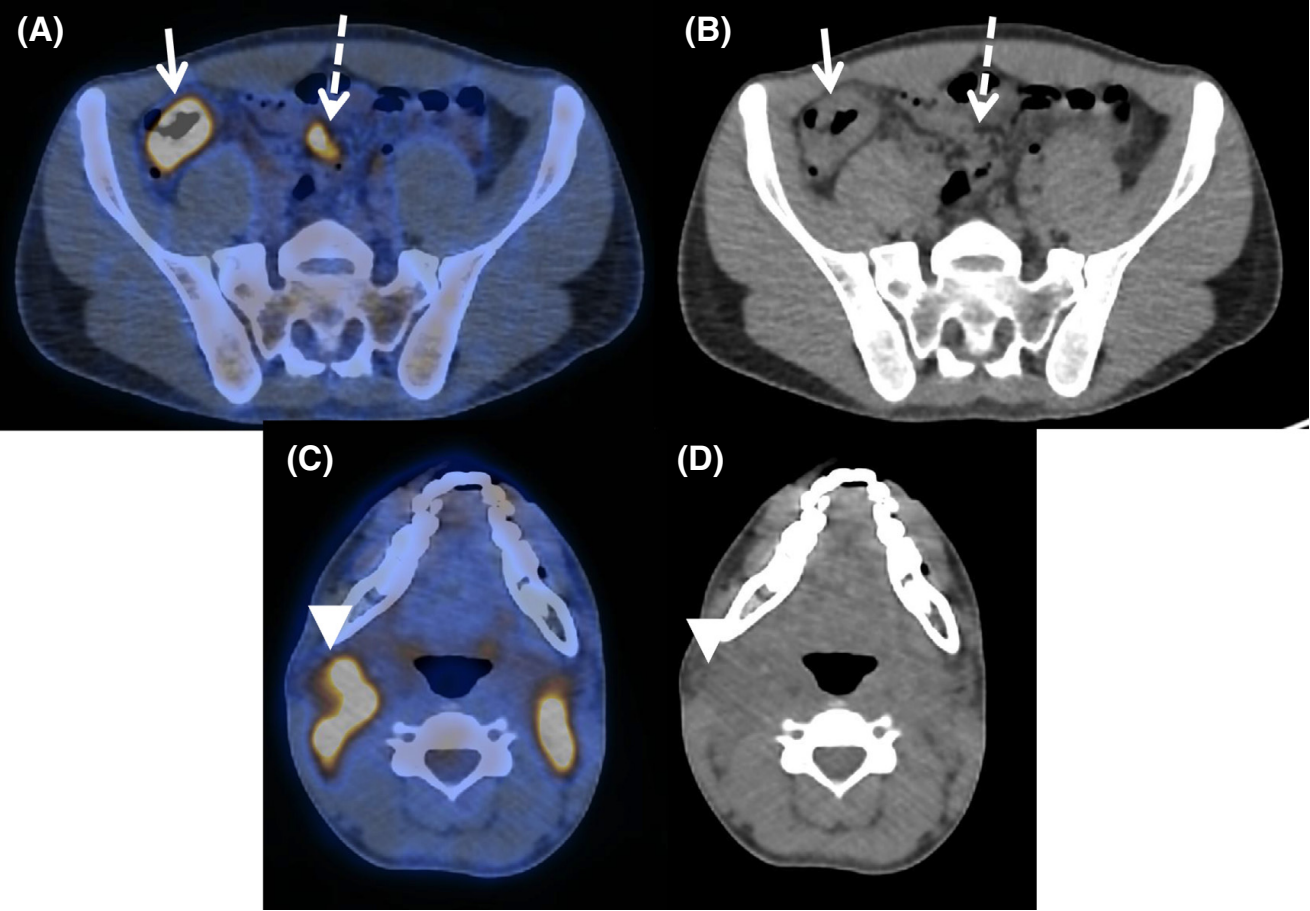
(A, C) ^18^F‐FDG PET/CT axial fused and (B, D) CT scan image of initial Hodgkin lymphoma staging. (A, B) Arrow shows colon tuberculosis localization (SUVmax 10.9), associated with mesenteric TB lymph nodes (dotted line) (SUVmax 6.6). (C, D) Arrowhead shows cervical lymph nodes of Hodgkin lymphoma, SUVmax 10.7. Measurement of SUVmax is inadequate to differentiate TB and HL disease.

Imaging with ^18^F‐FDG PET/CT is known to be the gold standard imaging for management, staging, restaging, assessment, and follow‐up of HL 1, 2, 3, 4, 5, 6. However, it is well known that ^18^F‐FDG PET/CT is a nonspecific tracer that can accumulate not only in tumor cells but also in macrophages, granulation, and inflammatory tissues. It allows assessment of both tumoral metabolism and inflammatory process. Tuberculosis extension often affects lymph nodes; hence, both TB and HL present with intense accumulation of FDG in the PET/CT imaging 7, and the measurement of SUVmax is inadequate to differentiate them (Fig. 2). As a consequence, TB lymph nodes mimicking HL make accurate HL stating difficult. Cases of TB mimicking lymphoma on ^18^F‐FDG PET/CT evaluation have been described 8, 9. Our case points out the complex intricacy of both TB and HL. Biopsy and pathology of all sites are not achievable, and metabolic features give valuable information in those situations.

Overlap of different conditions involving lymph nodes extension is challenging for accurate lymphoma staging.

^18^F‐FDG PET/CT appears to be a useful tool as problem solver in ambiguous situation: (1) guiding biopsy – as performed in our case with the colon pathological uptake proven to be tuberculosis; (2) allowing early treatment assessment – as with antituberculosis treatment initiation – with the benefit that rapid metabolic changes allow accurate management, whereas anatomical features alteration can be delayed; (3) giving accurate staging for adapted treatment; and (4) providing early and end‐of‐treatment prognosis of HL management.

## Authorship

NJ: performed PET examination, wrote the manuscript, and submitted case report. TCM and ML: performed PET examination and provided images. AVS: performed pathology analysis and provided pathology images. MB and FK: managed the patient and performed literature review. All authors reviewed and approved the final version of the manuscript.

## Conflict of Interest

None declared.
